# Myocardial Rupture following Carbon Monoxide Poisoning

**DOI:** 10.1155/2014/281701

**Published:** 2014-07-09

**Authors:** Gabija Dragelytė, Jūris Plenta, Sigitas Chmieliauskas, Algimantas Jasulaitis, Romas Raudys, Tomas Jovaiša, Robertas Badaras

**Affiliations:** ^1^Clinic of Anaesthesiology and Intensive Care, Centre of Toxicology, Faculty of Medicine, Vilnius University, Siltnamiu Street 29, LT-04130 Vilnius, Lithuania; ^2^Department of Pathology, Forensic Medicine and Pharmacology, Faculty of Medicine, Vilnius University, M. K. Čiurlionio Street 21/27, LT-03101 Vilnius, Lithuania; ^3^State Forensic Medicine Service under the Ministry of Justice of the Republic of Lithuania, Didlaukio Street 86E, LT-08303 Vilnius, Lithuania; ^4^Lithuanian University of Health Sciences, Eiveniu Street 2, LT-50009 Kaunas, Lithuania

## Abstract

We present the first case of severe cardiotoxicity of carbon monoxide leading to myocardial rupture and fatal outcome. 83-year-old woman was hospitalized 4 hours after the fire in her house with no respiratory or cardiac symptoms. After two days, she has suffered sudden collapse leading to cardiac arrest. Postmortem examination revealed intramural haemorrhage with myocardial rupture at the apex of the left ventricle. Minimal stenosis was noted in the proximal coronary arteries with no evidence of distal occlusion or any other long-standing heart disease. This case supports recommendations for targeted cardiovascular investigations in cases of CO poisoning.

## 1. Case Presentation

83-year-old woman was hospitalized 4 hours after the fire in her house, which she attempted to extinguish herself. Shortly afterwards, her son noticed persistent restlessness and agitation and therefore brought her to the Accident and Emergency Department in his own car. Prior to the incident, patient had no significant comorbidities or complaints. She was a nonsmoker, had no regular prescriptions, was living alone, was self-caring, and managed all the activities of daily life without assistance.

On admission, patient was conscious, alert, and oriented (Glasgow Coma Scale 15/15). Respiratory rate was 20/min, and lung sounds were clear on auscultation in all fields. ECG showed sinus rhythm, heart rate of 80/min, and no evidence of myocardial ischaemia. Arterial blood gas revealed pH-7,36, pCO2-35,9, pO2 98,5, HCO3-20,1, and COHb 15.4% (analysis performed on Radiometer ABL 835). At that time, patient was receiving supplementary oxygen at 5 L/min via Hudson mask with estimated inspiratory oxygen concentration of 40%. Due to low COHb on admission, patient did not meet the criteria for urgent hyperbaric oxygen. Patient was treated with oxygen, intravenous fluids, and empirical antibiotics.

Patient's respiratory function deteriorated on the second day of admission due to bilateral pneumonia. She was transferred to intensive care unit (ICU) and received positive pressure ventilation via endotracheal tube. Chest X-ray did not show any evidence of diffuse pulmonary infiltrates or oedema. Oxygen saturation was maintained above 95% with inspired oxygen concentration of up to 60%. Her condition remained stable and she did not require any other organ support for the following 24 hours. 12-lead ECG was done on admission to ICU and continuous ECG monitoring in standard lead II was used; however, it did not demonstrate any evidence of myocardial ischaemia until the following day when patient has suffered sudden cardiovascular collapse. She became progressively hypotensive and suffered asystolic cardiac arrest and all resuscitation attempts were unsuccessful. In our institution, cardiac enzyme tests are performed 6 hours after the signs of ischaemia; unfortunately, in this case, fulminant progression of cardiac pathology did not allow us to perform the tests.

## 2. Postmortem Report 

**Figure 1:**
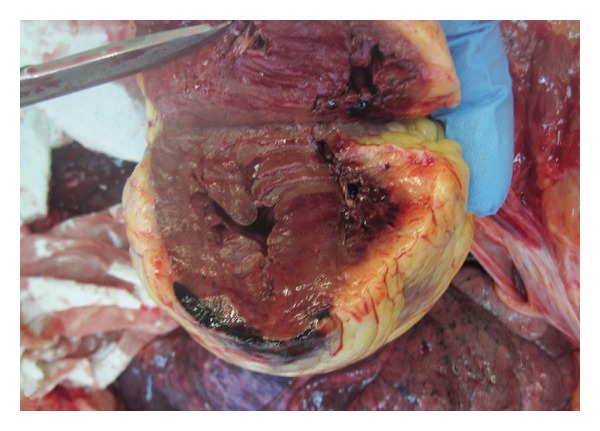
Transmural rupture.

First degree burns on the nose were noted on external examination; there were no further external injuries. Internal examination of the heart revealed distended pericardium, extensive intramural haemorrhage extending along the anterior interventricular artery from 2 cm below the branching off, all the way to the apex, measuring 4.5 × 4 cm with myocardial rupture at the apex of the left ventricle (Figure 1), and 450 mL of blood and clots in the pericardial sack. Heart weight was 410 grams, within the normal range. Atherosclerotic plaques were noted in the proximal coronary arteries with minimal stenosis. There was no evidence of distal coronary disease. Histological examination demonstrated damaged myocardial fibres and loss of myocardial tissue structure in the peri-rupture area. Minor atherosclerotic changes were noted in aorta. Mucosa of the airway appeared oedematous, with focal black soot staining. Hyperaemia was noted in the distal bronchi as well as thick light green secretions. Black soot was noted in some of the distal bronchi. No further macroscopic or microscopic injuries were noted.

Diagnosis following postmortem was as follows: acute poisoning with carbon monoxide and other products of combustion. Complications were myocardial infarction with myocardial rupture and pericardial tamponade. Concomitant disease was first degree burns of the nose and upper airway.

## 3. Discussion

Reported case occurred following the fire in a limited section of a traditional timber house with minimal presence of other substances or building materials; hence, products usually present in the wood smoke are likely causative agents. These include carbon and nitrogen oxides, volatile organic compounds, methane, aldehydes, benzenes, and a number of other substances in very small concentrations. While some of those are known to be cancerogenic or cause chronic cardiopulmonary effects, carbon monoxide is by far the most abundant substance with well-recognised mechanisms of acute toxicity. It is most likely that primary causative factor of myocardial rupture was toxicity of CO.

Cardiac effects of CO are caused by dual toxicity—hypoxia due to COHb formation and direct effect on myocardium. CO affinity to myoglobin is 60% higher than that of oxygen. Tissue hypoxia is produced by competitive binding on the oxygen-carrying hemoproteins (haemoglobin and myoglobin); mitochondrial respiration is impaired by binding to the cytochrome oxidase complex [1, 2]. Toxic effects result in arrhythmias, ventricular dysfunction, and myocardial infarction [3]. CO poisoning may cause angina and myocardial injury even in young patients with no preexisting coronary disease. Patients with ischaemic heart disease may have symptoms of angina with COHb levels as low as 5–10% [4]. In addition to direct effects, CO also causes nitrous oxide release, vascular smooth muscle relaxation, and hypotension [5].

In our opinion, preexisting cardiovascular condition was unlikely to make significant contribution to the fatal myocardial infarction. Patient had no significant comorbidities and was in good physical condition, with no history of ischaemic heart disease or complaints or treatment for it before admission. Postmortem results demonstrated minor atherosclerotic changes that are typical in this age group, likely to be fully compensated and asymptomatic. ECG on admission did not show any evidence of ischaemia. She had no history of preexisting hypertension, and neither clinical findings nor postmortem results demonstrated typical signs of long-standing hypertensive disease.

Patient has suffered thermal airway and lung injury which progressed to bilateral pneumonia. This was noted clinically and later proven by postmortem. Lung condition was identified early and promptly treated with endotracheal intubation and mechanical lung ventilation. Review of case history did not demonstrate clinically relevant hypoxia during her hospital stay. Patient was likely to suffer short hypoxic period during the fire. COHb level on admission was 15.4%, and afterwards she was breathing atmospheric air for 4 hours. Considering the CO elimination half-life of 4–6 hours in atmospheric air [6], it is possible that peak concentrations at the time were in the range of 20–30%. However, on admission, patient had no signs of acute cardiovascular toxicity.

In our opinion, it was direct myocardial toxicity of CO that caused severe myocardial infarction, further complicated by rupture and pericardial tamponade. Our case demonstrates that continuous ECG monitoring in standard lead II only may not be sensitive enough to pick up progression of myocardial damage. IV sedation on ITU masks possible symptoms of angina as well. It has been recommended previously that all patients should have 12-lead ECG and cardiac enzyme tests [7]. Based on this report, we would suggest performing cardiac enzyme tests electively even in the absence of ECG changes. An echocardiography is recommended if initial tests suggest myocardial ischaemia [8]. This could lead to early diagnosis and timely management of cardiac injury.
